# Tobacco control in latin america: achievements, gaps and new challenges

**DOI:** 10.17843/rpmesp.2023.403.13284

**Published:** 2023-09-27

**Authors:** Andrea Alcaraz, Andrés Pichon-Riviere

**Affiliations:** 1 Institute for Clinical and Health Effectiveness (IECS), Department of Health Technology Assessment and Economic Evaluations, Buenos Aires, Argentina. Institute for Clinical and Health Effectiveness (IECS) Department of Health Technology Assessment and Economic Evaluations Buenos Aires Argentina; 2 Epidemiology and Public Health Research Center - National Council for Scientific and Technical Research (CIESP-CONICET), Buenos Aires, Argentina. Epidemiology and Public Health Research Center National Council for Scientific and Technical Research (CIESP-CONICET) Buenos Aires Argentina

Smoking is one of the great epidemics that has endured since the 20th century. Like many epidemics, it is not easy to overcome. At times we manage to halt its growth, only to discover that it has become endemic and more difficult to eradicate. Moreover, it adapts and transforms, circumventing legislation, using new marketing strategies, with innovations such as the electronic cigarette, or spreading to populations that until recently had remained relatively untouched, such as women and adolescents. As with any epidemic, only in-depth knowledge will enable us to develop the tools for its definitive eradication. In this editorial, we review what we know so far, what has been done well, what remains to be done, and what new challenges we will have to face.

## What we know

Tobacco use causes more than 8 million deaths per year worldwide, 7 million from direct consumption and 1.2 million from passive smoking ^1^. This makes it one of the greatest public health challenges. Smoking is a preventable risk factor associated with numerous diseases such as cardiovascular disease, cerebrovascular disease, diabetes, chronic obstructive pulmonary disease, lung cancer and at least 10 other types of cancer ^1^.

In Latin America, smoking is one of the main risk factors for death and disability, contributing to poverty and exerting an enormous economic burden on health systems. A study conducted in eight Latin American countries, representing 80% of the region’s population, estimated that smoking is responsible each year for 35,000 deaths, 2.25 million episodes of related diseases, 12.2 million years of healthy life lost, US$ 22.8 billion in direct medical costs, US$ 16.2 billion in lost productivity and US$ 10.8 billion in caregiver costs. These economic losses represent 1.4% of the aggregate gross domestic product of the countries in the region ^2^.

## What we are doing well

The Region of the Americas experienced a decrease in the prevalence of current tobacco use, from 28% in 2000 to 16.3% in 2020, although with a large variability from 29.2% consumption in Chile to 5.0% in Panama ^3^. Unfortunately, this reduction has not prevented tobacco use in Latin America from advancing more rapidly among women than in any other part of the world, reducing the gap between men and women: prevalence is 21.3% in men and 11.3% in women, compared to the global prevalence of 36.7% men and 7.8% women ^3^.

One of the most notable achievements in the region is that almost all Latin American countries have ratified the World Health Organization (WHO) Framework Convention on Tobacco Control, demonstrating a significant political commitment to address tobacco control.

The “MPOWER” measures, established by WHO in 2008, are a comprehensive set of strategies to address tobacco control globally that aim to help countries implement the measures under the Framework Convention. More than 70% of the world’s population, or 5600 million people, are covered by at least one MPOWER measure at the highest level of compliance, while only 4 countries have already achieved the full package of MPOWER measures ^1^.

Significant progress has been made in the Americas region regarding the implementation of MPOWER measures, with 26 of the 35 countries achieving the highest level of implementation of at least one measure, equivalent to a population coverage of 96% ^3^. In a notable milestone in 2020, South America became the first sub-region in the Americas to declare itself smoke-free, implying a total ban on smoking in public places, enclosed work environments and public transport systems ^3^. By 2025, the Region of the Americas is projected to reach a tobacco use prevalence of 14.9%, which puts it on track to achieve target 5 of the Global Action Plan for the Prevention and Control of Noncommunicable Diseases 2013-2020, i.e., a 30% relative reduction in the prevalence of tobacco use among persons aged 15 years and older ^3^.

## What we are lacking

According to the Pan American Health Organization (PAHO) Report on Tobacco Control for the Region of the Americas 2022, of the 35 countries in the region, only 10 countries have surveillance systems that provide recent, periodic and representative data on adult and youth tobacco use; 24 countries have implemented measures aimed at protecting the population from exposure to indirect tobacco smoke; only 6 countries offer comprehensive smoking cessation programs; 22 countries have adopted large graphic warnings that provide information on the dangers of smoking on tobacco product packages, but only Uruguay has implemented plain packaging; only 9 countries have total bans on tobacco product advertising, promotion, and sponsorship; and only 3 countries apply indirect taxes representing 75% or more of the retail price of cigarettes ^1^^,^^3^.

Despite advances in data collection on tobacco prevalence, we still face significant challenges in obtaining detailed information on tobacco use in population subgroups, such as young and indigenous communities, as well as data disaggregated by gender and income level. This information is crucial for designing targeted strategies to address the needs of these groups and reduce disparities regarding tobacco prevalence.

Young people (13-15 years) have different smoking patterns, with an average consumption of 11.3%, higher than the world average of 10.3%, reaching 25.3% in Dominica. E-cigarette use is more frequent in this population with enormous variation between countries ^3^.

In addition, we lack up-to-date data on secondhand smoke exposure in different settings, making it difficult to accurately assess the impact of smoke-free policies.

There is an important gap in the access to tobacco cessation services, such as telephone helplines, medications, and behavioral therapies.

We must strengthen restrictions on tobacco advertising and promotion, particularly online and on social media, in which the youth are vulnerable to exposure to pro-tobacco messages.

Despite increases in tobacco taxes, cigarette prices in the region remain relatively low. Only 3 countries have reached the minimum target set by WHO, despite being the most cost-effective measure in the MPOWER package. Steadily increasing taxes is an effective strategy to discourage tobacco use and generate revenue for health care and prevention.

A study conducted by universities, ministries and academic institutions in eight Latin American countries estimated that full implementation and enforcement of the four strategies: taxation, plain packaging, advertising bans and smoke-free environments would prevent 27,000, 78,000, 71,000 and 39,000 deaths, respectively, over the next 10 years, and would represent a total of $9.32 billion in economic benefits ^2^.

On the other hand, there is little global information on the climate impact of crops and wastes produced by tobacco.

## New challenges

Many forms of tobacco use are advancing rapidly worldwide, including electronic cigarettes, smokeless tobacco products, cigars, cigarettes, hookahs, bidis, kreteks, menthol cigarettes and other smokeless forms of tobacco. These products are associated with different issues such as nicotine addiction, carcinogens and health problems. Young people are particularly prone to these new forms of consumption ^4^. PAHO warns about the increasing availability and accessibility of novel and emerging nicotine and tobacco products, which represents a significant threat to tobacco control efforts. It also warns that the tobacco industry employs deceptive tactics to attract consumers and expand its markets. PAHO/WHO urges governments to implement regulations to prevent non-smokers from starting to use these products, to avoid the re-normalization of tobacco use in society, and to safeguard the well-being of future generations. Currently, the sale of electronic nicotine delivery systems is banned in seven countries in the Americas, while thirteen other countries have adopted partial measures limiting their use, advertising, promotion and sponsorship or requiring warnings on the packaging of these products ^3^. Unfortunately, fifteen countries have not yet established any regulatory framework ^3^.

In conclusion, Latin America has made significant progress in tobacco control, but still faces important challenges. It is critical to continue to collect accurate data, address inequalities in tobacco use, and strengthen existing policies and programs. Smoking is a huge public health problem, and requires ongoing commitment from governments, health organizations and the society as a whole to reduce its impact in our region.
