# Supplementary material for: Tobacco control in latin america: achievements, gaps and new challenges
Source: Rev Peru Med Exp Salud Publica. 2023 Sep 27;40(3):264–6. doi: 10.17843/rpmesp.2023.403.13284 (PMC10953674; doi:10.17843/rpmesp.2023.403.13284)
Supplement: Translation

# Translation: El control del tabaco en América Latina: logros, pendientes y nuevos desafíos

**Authors:** Andrea Alcaraz, Andrés Pichon-Riviere

**Affiliations:** 1 Instituto de Efectividad Clínica y Sanitaria (IECS), Departamento de Evaluación de Tecnologías Sanitarias y Evaluaciones Económicas, Buenos Aires, Argentina.; 2 Centro de Investigaciones en Epidemiología y Salud Pública - Consejo Nacional de Investigaciones Científicas y Técnicas (CIESP-CONICET), Buenos Aires, Argentina.

El tabaquismo es una de las grandes epidemias que ha perdurado desde el siglo XX. Como muchas epidemias, no es fácil de doblegar. A veces logramos detener su crecimiento, solo para descubrir que se ha vuelto endémica y difícil de erradicar. Además, se adapta y transforma, sorteando legislaciones, utilizando nuevas estrategias de marketing, con innovaciones como el cigarrillo electrónico, o avanzando sobre poblaciones que hasta hace poco se habían mantenido relativamente al margen, como las mujeres y los adolescentes. Como en cualquier epidemia, solo un conocimiento profundo nos permitirá desarrollar las herramientas para su erradicación definitiva. En esta editorial, repasamos que es lo que sabemos hasta ahora, que se ha hecho bien, lo que falta por hacer, y cuáles son los nuevos desafíos que deberemos enfrentar.

## Lo que sabemos

El uso de tabaco causa más de 8 millones de muertes al año en todo el mundo, 7 millones por consumo directo y 1,2 millones por tabaquismo pasivo^1^. Esto lo convierte en uno de los mayores desafíos para la salud pública. El tabaquismo es un factor de riesgo evitable que se asocia a numerosas enfermedades como enfermedades cardiovasculares, cerebrovasculares, diabetes, enfermedad pulmonar obstructiva crónica, cáncer de pulmón y al menos otras 10 neoplasias ^1^.

En América Latina, es uno de los principales factores de riesgo de muerte y discapacidad, contribuyendo a la pobreza y ejerciendo una enorme carga económica a los sistemas de salud. Un estudio realizado en ocho países latinoamericanos que representan el 80% de la población de la región, estimó que el tabaquismo es responsable cada año de 351000 muertes, 2,25 millones de episodios de enfermedades relacionadas, 12,2 millones de años de vida saludable perdidos, 22800 millones de dólares en costos médicos directos, 16200 millones de dólares en pérdida de productividad y 10800 millones de dólares en costos de cuidadores. Estas pérdidas económicas representan el 1,4% del producto bruto interno agregado de los países de la región ^2^.

## Lo que estamos haciendo bien

La Región de las Américas registró una disminución de la prevalencia del consumo actual de tabaco, que pasó de 28% en el año 2000 al 16,3% en el 2020 aunque con una gran variabilidad desde un 29,2% de consumo en Chile a un 5,0% en Panamá^3^. Lamentablemente esta reducción no ha impedido que en América Latina, el consumo de tabaco haya avanzado entre las mujeres más rápidamente que en cualquier otra parte del mundo, reduciendo la brecha entre hombres y mujeres: la prevalencia es del 21,3% en hombres y del 11,3% en mujeres, en comparación con la prevalencia mundial de 36,7% hombres y 7,8% mujeres ^3^.

Uno de los logros más destacados en la región es que casi todos los países latinoamericanos han ratificado el Convenio Marco para el Control del Tabaco de la Organización Mundial de la Salud (OMS), lo que demuestra un compromiso político importante para abordar el problema del tabaquismo.

Las medidas «MPOWER» establecidas por la OMS en 2008 para ayudar a los países a aplicar medidas bajo el Convenio Marco representan un conjunto integral de estrategias para abordar el control del tabaco a nivel global. Más del 70% de la población mundial, es decir, 5600 millones de personas, está cubierta por al menos una medida MPOWER al más alto nivel de cumplimiento, mientras que solo 4 países han alcanzado ya el paquete completo de medidas MPOWER ^1^.

En la región de las Américas, se han experimentado avances significativos en la implementación de medidas MPOWER, con 26 de los 35 países que han logrado el mayor nivel de aplicación de al menos una medida, lo que equivale a una cobertura poblacional del 96% ^3^. En un hito notable en 2020, Sudamérica se convirtió en la primera subregión de las Américas en declararse libre de humo, lo que implica una prohibición total del tabaquismo en lugares públicos, entornos laborales cerrados y sistemas de transporte público ^3^. Se proyecta que, para el año 2025, la Región de las Américas alcance una prevalencia del consumo de tabaco del 14,9%, lo que la encamina a alcanzar la meta 5 del Plan de acción mundial para la prevención y el control de las enfermedades no transmisibles 2013-2020, es decir, una reducción relativa del 30% en la prevalencia del consumo de tabaco entre personas de 15 años o más ^3^.

## Lo que nos falta

Según el Informe sobre el control del tabaco para la Región de las Américas 2022 de la Organización Panamericana de la Salud (OPS), de los 35 países de la región únicamente 10 países cuentan con sistemas de vigilancia que proporcionan datos recientes, periódicos y representativos sobre el consumo de tabaco en adultos y jóvenes; 24 países han implementado medidas destinadas a proteger a la población de la exposición al humo de tabaco indirecto; tan solo 6 países ofrecen programas integrales para la cesación del tabaquismo; 22 países han adoptado advertencias graficas grandes que informan sobre los peligros de fumar en los paquetes de productos de tabaco pero sólo Uruguay ha implementado el empaquetado plano; solo 9 países tienen prohibiciones totales sobre la publicidad, promoción y patrocinio de productos de tabaco; y únicamente 3 países aplican impuestos indirectos que representan el 75% o más del precio de venta minorista de los cigarrillos ^1^^,^^3^.

A pesar de los avances en la recopilación de datos sobre la prevalencia del tabaco, todavía enfrentamos desafíos importantes en la obtención de información detallada sobre el consumo de tabaco en subgrupos de población, como los jóvenes y las comunidades indígenas, y de datos discriminados por género y nivel de ingresos. Esta información es crucial para diseñar estrategias específicas que aborden las necesidades de estos grupos y reduzcan las disparidades en la prevalencia del tabaco.

La población joven (de 13 a 15 años) tiene patrones diferentes de uso siendo el consumo promedio del 11,3%, superior al promedio mundial del 10,3%, llegando hasta el 25,3% en Dominica. El uso del cigarrillo electrónico es más frecuente en esta población con una enorme variabilidad entre los países ^3^.

Además, carecemos de datos actualizados sobre la exposición al humo de segunda mano en diversos entornos, lo que dificulta la evaluación precisa del impacto de las políticas de espacios libres de humo. Queda una deuda pendiente a la hora de ampliar el acceso a servicios de cesación del tabaco, como líneas telefónicas de ayuda, medicamentos y terapias conductuales.

Debemos fortalecer las restricciones a la publicidad y promoción del tabaco, especialmente en línea y en las redes sociales, donde los jóvenes son especialmente vulnerables a la exposición a mensajes pro-tabaco.

A pesar de los aumentos en los impuestos al tabaco, los precios de los cigarrillos en la región siguen siendo relativamente bajos. Solo 3 países han alcanzado la meta mínima establecida por la OMS, a pesar de ser la medida más costo-efectiva del paquete MPOWER. Incrementar los impuestos de manera constante es una estrategia eficaz para desalentar el consumo de tabaco y generar ingresos para la atención médica y la prevención.

Un estudio realizado por universidades, ministerios y entidades académicas de ocho países de Latinoamérica estimó que la plena aplicación y cumplimiento de las cuatro estrategias: impuestos, empaquetado plano, prohibición de la publicidad y entornos libres de humo evitaría 271000, 78000, 71000 y 39000 muertes, respectivamente, en los próximos 10 años, y supondría un total de 93200 millones de dólares de beneficios económicos ^2^.

Por otra parte, hay escasa información a nivel global del impacto climático de los cultivos y desechos generados por el tabaco.

## Nuevos desafíos

Diversas formas de consumir tabaco avanzan rápidamente a nivel mundial abarcando cigarrillos electrónicos, productos de tabaco sin combustión, puros, cigarritos, narguiles, bidis, kreteks, cigarrillos mentolados y las formas de tabaco sin humo. Estos productos se asocian con diferentes problemáticas como la adicción a la nicotina, los carcinógenos y los problemas de salud. Los jóvenes son particularmente proclives a estas nuevas formas de consumo ^4^. La OPS advierte sobre la creciente disponibilidad y accesibilidad de productos de nicotina y tabaco novedosos y emergentes, lo que representa una amenaza significativa para los esfuerzos de control del tabaco. Además, alerta que la industria tabacalera emplea tácticas engañosas para atraer consumidores y expandir sus mercados. La OPS/OMS insta a los gobiernos a implementar regulaciones para prevenir que las personas no fumadoras comiencen a utilizar estos productos, evitar que el consumo de tabaco vuelva a normalizarse en la sociedad y salvaguardar el bienestar de las generaciones venideras. En la actualidad, la venta de sistemas electrónicos de administración de nicotina está prohibida en siete países de las Américas, mientras que otros trece países han adoptado medidas parciales que limitan su uso, publicidad, promoción y patrocinio o requieren advertencias en el empaquetado de estos productos ^(^^3^. Lamentablemente, quince países aún no han establecido ningún marco regulador ^(^^3^.

En resumen, América Latina ha avanzado significativamente en el control del tabaco, pero aún enfrenta desafíos importantes. Es fundamental seguir recopilando datos precisos, abordar las desigualdades en el consumo de tabaco y fortalecer las políticas y programas existentes. El tabaquismo es un enorme problema de salud pública, y requiere un compromiso continuo de gobiernos, organizaciones de salud y la sociedad en su conjunto para reducir su impacto en nuestra región.
